# Cerebral Venous Thrombosis and Its Clinical Diversity

**DOI:** 10.7759/cureus.14750

**Published:** 2021-04-29

**Authors:** Giovana Ennis, Nelson Domingues, Joana Silva Marques, Pedro Ribeiro, Cristina Andrade

**Affiliations:** 1 Department of Internal Medicine, Centro Hospitalar Tondela-Viseu, Viseu, PRT

**Keywords:** immune thrombocytopenia purpura, cerebral venous thrombosis, thrombocytopenia, stroke, cerebral venous infarction

## Abstract

Cerebral venous thrombosis (CVT) is a serious medical condition which is difficult to diagnose because of its wide range of clinical presentations. The symptoms can vary from an isolated headache to coma. Here, we present the case of a 76-year-old female patient with a history of immune thrombocytopenic purpura, arterial hypertension, and pulmonary embolism. The diagnosis of CVT was challenging because the initial form of disease presentation mimicked a transient ischemic attack (transient aphasia and right hemiparesis). Therapeutical decisions were also a challenge because, at the time of the diagnosis, the patient was suffering from severe thrombocytopenia (29 × 10^9^/L), which had to be taken into account. After multidisciplinary discussions, therapeutic subcutaneous enoxaparin was started, resulting in a progressive and significant neurological recovery. In presenting this case, our primary goal is to point out that CVT can be difficult to diagnose because of its wide range of clinical presentations. Headache (a symptom that was never present in this case) is the most frequent complaint, occurring in 90% of cases. Following diagnosis, an etiological study is required.

## Introduction

Cerebral venous thrombosis (CVT) is a rare [1] and serious medical condition. Symptoms can vary from an isolated headache to coma [2]. Although CVT incidence in Portugal has been estimated at 0.22/100,000 per year [3], recognition and diagnosis have grown owing to increasing clinical awareness. The diagnosis and treatment of CVT should be considered an emergency because complete clinical recovery is possible[4].

This article was previously presented as an oral presentation at the 10th World Congress on Controversies in Neurology on March 17-20, 2016.

## Case presentation

A 76-year-old female patient with immune thrombocytopenic purpura (ITP), diagnosed eight years earlier, treated at the time with prednisolone 5 mg/day, attended the Emergency Department (ED) complaining of periods of amnesia and lethargy that had started a week earlier and were becoming more frequent. The patient also reported multiple episodes of transient right hemiparesis in the last two days, subsiding in one to two hours. She denied having a headache, fever, or involuntary movements. The patient also reported a personal history of arterial hypertension, atrial fibrillation (AF), hypothyroidism, and pulmonary embolism. On examination, the patient revealed 2/5 (Medical Research Council) right-sided hemiparesis and global aphasia, with a National Institutes of Health Stroke Scale (NIHSS) of 10, and no signs of meningeal irritation. The initial study was remarkable only for severe thrombocytopenia (platelets: 56 × 10^9^/L). The patient underwent cerebral computed tomography (CT) that showed no signs of ischemic, hemorrhagic vascular event or any space-occupying lesion. She was re-evaluated after having undergone these examinations, and, surprisingly, aphasia had disappeared, but a slight (4/5) right hemiparesis remained. Five hours after admission, her clinical status worsened, again presenting global aphasia and 3/5 right hemiparesis (NIHSS: 8). At this point, it was decided to perform a CT angiography (CTA), but no images revealing intracranial arterial occlusion or stenosis were identified. This intriguing case was discussed with a multidisciplinary team, where several diagnostic hypotheses were suggested. At this point of the investigation, the first hypothesis was a transient ischemic attack due to carotid stenosis or embolism (given the repetition of the same pattern: aphasia and right-sided hemiparesis). A cervical Doppler done later in the investigation ruled out carotid or vertebral stenosis. Cardioembolic etiology could also be considered as the patient also had AF and was not anti-coagulated. However, the same pattern of symptoms in multiple episodes described that there would have to be embolization to the same territory, which was implausible.

Another hypothesis was epilepsy, with periods of altered state of consciousness corresponding to post-ictal events. The absence of involuntary or tonic-clonic generalized movements led us to put this hypothesis in second place at the time. The unavailability of electroencephalogram in the ED made it impossible to immediately exclude this hypothesis.

A lumbar puncture was delayed as the hypothesis of encephalitis, while plausible, was remote because there were no strong clinical signs to support the diagnosis (absence of fever and meningeal signs), and because of the risk of procedure-related complications owing to the thrombocytopenia. Given the normal cerebral CT, cerebral magnetic resonance imaging might have added diagnostic value at this stage but was unavailable. The patient was therefore admitted to the Stroke Unit for observation and further study.

**Figure 1:**
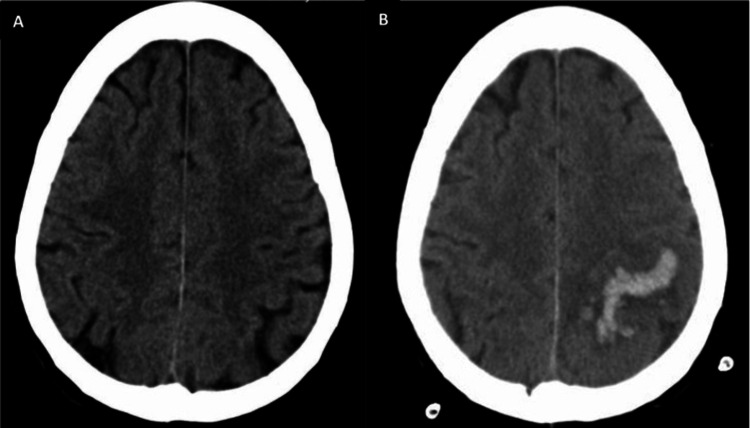
Cerebral CT findings. Initial cerebral CT showing no sign of any ischemic or hemorrhagic vascular event or any space-occupying lesion (A). Cerebral CT revealing a vascular lesion with a left rolandic cortico-subcortical hemorrhagic component and reduced amplitude of the regional sulci (B) CT: computed tomography

On the first day, the patient suffered three tonic-clonic seizures, with involuntary movements being more pronounced in the right limbs. Approximately 12 hours later, a new cerebral CT revealed a vascular lesion with a left rolandic cortico-subcortical hemorrhagic component and reduced amplitude of the regional sulci (Figure 1).

**Figure 2:**
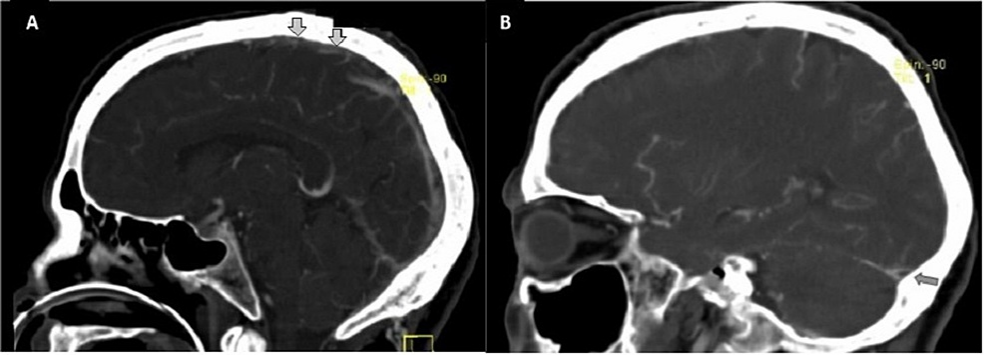
Cerebral veno-CT findings. Cerebral veno-CT confirmed the absence of filling of the superior longitudinal sinus (A) and the right lateral sinus (B) CT: computed tomography

Due to the relatively high density of the medial third of the superior longitudinal sinus cerebral veno-CT was conducted, which confirmed the absence of filling of the superior longitudinal sinus and the right lateral sinus; the presence of the left frontoparietal intraparenchymal lesion was suggestive of venous infarction (Figure 2).

Therapeutical decisions were also a challenge because, at the time of the diagnosis, the patient was suffering from severe thrombocytopenia (29 × 10^9^/L). After multidisciplinary discussions, therapeutic subcutaneous enoxaparin (60 mg twice daily) was started, resulting in progressive and significant neurological recovery. Later, parenteral anticoagulant was switched to warfarin.

A hematology specialist confirmed the diagnosis of ITP, judging that additional study or escalation of therapy (third-line drugs such as rituximab or thrombopoietin receptor agonists) was unnecessary because the patient consistently had platelets in excess of 30 × 10^9^/L with no history of bleeding [5] and usually responded well to corticoid therapy, which was increased to 0.5 mg/kg/day of prednisolone, improving her thrombocytopenia (platelets 135 × 10^9^/L).

## Discussion

In this patient, the presence of thrombocytopenia associated with CVT led to the suggestion that these could be manifestations of a paraneoplasic syndrome or a connective tissue disease. A comprehensive study was performed that ruled out these hypotheses.

In this patient, the diagnosis was particularly challenging because the initial form of presentation and the absence of certain diagnostic tools led us to suspect a transient ischemic attack. Furthermore, as CVT usually occurs in young adults, the age of our patient also added to the complexity of the case. Although headache is the most frequent complaint, occurring in 90% of cases [6], the patient never displayed this symptom. The most common focal sign is mono or hemiparesis. Aphasia may also appear, especially if the lateral sinus is involved. Seizures are the initial form of presentation in 39% of cases [7]. There have also been reports of CVT presenting as transient ischemic attacks [8].

Following diagnosis, an etiological study is required, and the search for a hidden neoplasic disease should not be overlooked even when there is a plausible cause for CVT, especially in patients over 40 years of age. Treatment for patients with an initial unprovoked venous thromboembolic event consists of 6-12 months of anti-coagulation therapy, even in patients with intracranial hemorrhage [9]. Warfarin (international normalized ratio target of 2.0-3.0) and dabigatran 150 mg twice daily have similar effectiveness and safety. European guidelines published in 2017 do not recommend direct oral anti-coagulants for prevention of recurrent venous thrombosis after CVT.

In this case, CVT was the second venous thromboembolic event. Furthermore, as the patient also had AF, anti-coagulation was continued with warfarin. ITP increases the risk of both arterial and venous thrombosis, including CVT, because of the chronic inflammatory state associated with the disease, even with thrombocytopenia. ITP may be a manifestation of an evolving tissue disorder also associated with an increased risk of thrombosis [10].

## Conclusions

CVT can be hard to diagnose owing to its plethora of clinical presentations. Furthermore, it is usually of multifactorial cause; hence, physicians should search for more than one risk factor, even when there is a plausible cause for CVT.
